# Prediction of post-stroke motor recovery benefits from measures of sub-acute widespread network damages

**DOI:** 10.1093/braincomms/fcad055

**Published:** 2023-03-01

**Authors:** Cyprien Rivier, Maria Giulia Preti, Pierre Nicolo, Dimitri Van De Ville, Adrian G Guggisberg, Elvira Pirondini

**Affiliations:** Department of Radiology and Medical Informatics, Faculty of Medicine, University of Geneva, Geneva 1202, Switzerland; Division of Neurocritical Care and Emergency Neurology, Department of Neurology, Yale School of Medicine, New Haven, CT 06510, USA; Department of Radiology and Medical Informatics, Faculty of Medicine, University of Geneva, Geneva 1202, Switzerland; CIBM Center for Biomedical Imaging, Lausanne 1015, Switzerland; Medical Image Processing Laboratory, Neuro-X Institute, Ecole Polytechnique Fédérale de Lausanne (EPFL), Geneva 1202, Switzerland; University of Applied Sciences and Arts Western Switzerland, Delémont 2800, Switzerland; Medical Image Processing Laboratory, Neuro-X Institute, Ecole Polytechnique Fédérale de Lausanne (EPFL), Geneva 1202, Switzerland; CIBM Center for Biomedical Imaging, Lausanne 1015, Switzerland; Department of Radiology and Medical Informatics, Faculty of Medicine, University of Geneva, Geneva 1202, Switzerland; Universitäre Neurorehabilitation, University Hospital of Berne, Inselspital, Berne 3010, Switzerland; Division of Neurorehabilitation, Department of Clinical Neurosciences, University Hospital of Geneva, Geneva 1205, Switzerland; Department of Radiology and Medical Informatics, Faculty of Medicine, University of Geneva, Geneva 1202, Switzerland; Department of Physical Medicine and Rehabilitation, University of Pittsburgh, Pittsburgh, PA 15213, USA; Rehabilitation Neural Engineering Laboratories, University of Pittsburgh, Pittsburgh, PA 15213, USA; Department of BioEngineering, University of Pittsburgh, Pittsburgh, PA 15213, USA; Department of Neurological Surgery, University of Pittsburgh, Pittsburgh, PA 15213, USA; Department of Neurobiology, University of Pittsburgh, Pittsburgh, PA 15213, USA

**Keywords:** Stroke, motor recovery, prediction, brain connectivity

## Abstract

Following a stroke in regions of the brain responsible for motor activity, patients can lose their ability to control parts of their body. Over time, some patients recover almost completely, while others barely recover at all. It is known that lesion volume, initial motor impairment and cortico-spinal tract asymmetry significantly impact motor changes over time. Recent work suggested that disabilities arise not only from focal structural changes but also from widespread alterations in inter-regional connectivity. Models that consider damage to the entire network instead of only local structural alterations lead to a more accurate prediction of patients’ recovery. However, assessing white matter connections in stroke patients is challenging and time-consuming. Here, we evaluated in a data set of 37 patients whether we could predict upper extremity motor recovery from brain connectivity measures obtained by using the patient’s lesion mask to introduce virtual lesions in 60 healthy streamline tractography connectomes. This indirect estimation of the stroke impact on the whole brain connectome is more readily available than direct measures of structural connectivity obtained with magnetic resonance imaging. We added these measures to benchmark structural features, and we used a ridge regression regularization to predict motor recovery at 3 months post-injury. As hypothesized, accuracy in prediction significantly increased (*R*^2^ = 0.68) as compared to benchmark features (*R*^2^ = 0.38). This improved prediction of recovery could be beneficial to clinical care and might allow for a better choice of intervention.

## Teaser

Rivier *et al*. report that global measures of brain connectivity estimated by introducing virtual lesions in healthy structural connectomes significantly increase the accuracy of motor recovery prediction in stroke patients when added to focal and clinical measures used until now.

## Graphical Abstract

**Graphical Abstract:**
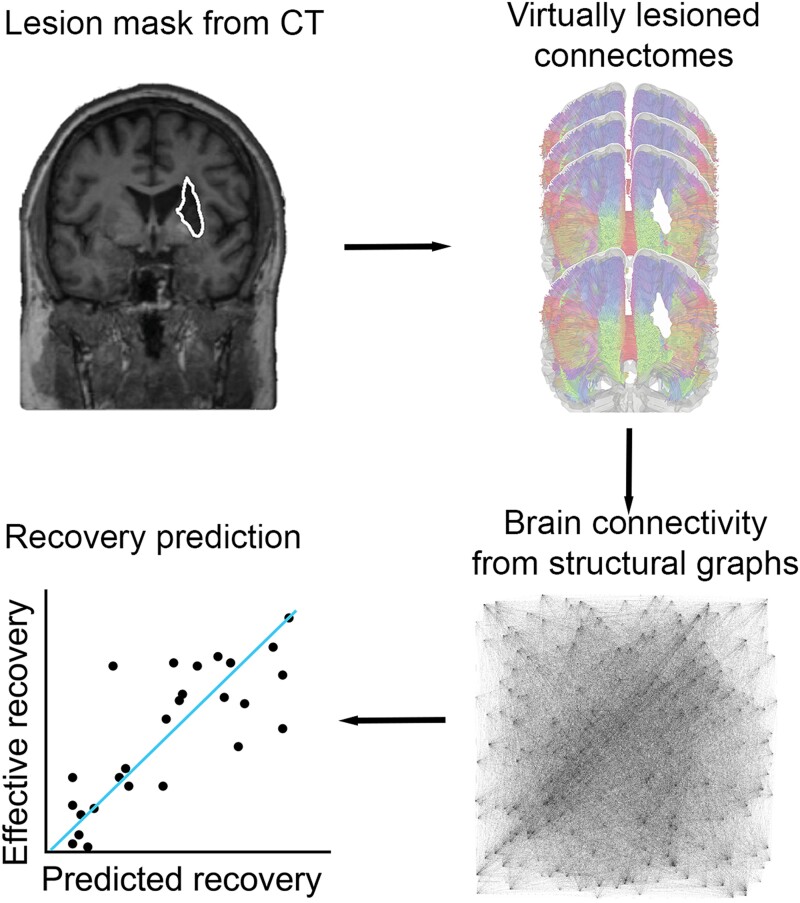

## Introduction

In the USA alone, almost 800 000 people are affected every year by a cerebral stroke with consequences that include severe motor, cognitive, or emotional handicap.^1^ Unfortunately, less than 15% of the patients achieve full recovery, making the number of persons currently living in the USA with motor impairments as a consequence of stroke on the order of millions.^1^ An additional challenge is the heterogeneity in outcome and individual recovery potential that strongly influence our ability to identify the optimal neurorehabilitative programme to maximize individual treatment outcome. Indeed, even experienced clinicians find it difficult to accurately predict patients’ level of recovery.^2^ Accurate prediction in the early stage after the lesion has the potential to yield a patient-specific recovery trajectory, stratify patients into recovery-focused clinical trials and guide rehabilitation strategies including discharge destination.^2^

It has already been shown that the severity of motor impairment at admission provides useful information on the recovery trajectory.^3^ Indeed, the comparison of the Fugl–Meyer Assessment (FMA) score obtained right after the stroke and 3–6 months later led to the postulation of the proportional recovery model,^4^ which states that the majority of patients recover on average 70% of their initial impairments. However, models that aim to predict patients’ percentage of initial impairment may lead to an overestimation of the fit between initial impairment and clinical recovery due to mathematical coupling and ceiling effects.^5,6^ Moreover, the 70% rule does not apply to all patients. Indeed, even though the recovery trajectory of about two-thirds of the patients fits this so-called proportional rule, the remaining ones, the so-called non-fitters, show very poor to no improvement.^7^ More recent work avoiding mathematical coupling and ceiling effects confirmed that patients present two distinct recovery patterns^8^ and that the clinical recovery shows proportionality to the initial impairment, although with much less explained variance.^9^ Prediction of motor recovery remains, thus, an important and currently unmet need.

The integrity of the cortico-spinal tract (CST), as assessed with brain imaging or with motor-evoked potentials, has also been proposed as a good predictor of motor recovery after stroke.^10,11^ Indeed, large CST lesions, as depicted by a high degree of CST asymmetry, are linked to poor motor improvement.^12,13^ Yet, a combination of clinical assessments and measures of CST integrity allow a reliable prediction of motor recovery in only 75% of patients.^10,11^

The lack of generalization of these prediction models might be explained by the use of measures derived purely from focal brain regions. Indeed, increasing evidence settled a new clinical concept in stroke: connectional *diaschisis*, which illustrates the idea that even a focal lesion might induce a loss of functionality in a territory that is distant to the lesion.^14^ This observation is not surprising if we consider the brain as one complex network where the different regions are nodes linked together by a global architecture that makes them dependent on one another.^15^

For this reason, recent studies attempted to include in the prediction models network measures extracted from functional^9,16–18^ and diffusion-weighted^10,18,19^ magnetic resonance imaging (MRI) with encouraging results. However, measuring functional and diffusion-weighted MRI signals requires long and expensive acquisitions that are not easily implementable in clinical settings particularly in the acute phase post-lesion.

Here we propose a novel method to combine focal structural damages and alterations in widespread network overcoming the disadvantage of individual functional and diffusion-weighted MRI acquisitions. Specifically, we embedded a patient’s lesion into a streamline tractography connectomes of 60 healthy subjects, thus indirectly estimating the stroke impact on the whole brain connectome. We constructed a structural graph between *N* = 360 atlas regions and with edges weighted to depict the number of white matter connections between cortical regions. We then computed weighted and unweighted graph measures that characterize network properties and used them to predict upper-limb motor recovery at 3 months post-lesion. When adding these measures to features previously used (lesion volume, initial motor impairment and CST asymmetry), the recovery prediction significantly improved, demonstrating the importance of considering widespread network dysfunctions for accurate and precise patients’ assessments.

## Materials and methods

### Participants

Thirty-seven subjects were included in this study (data set #1). Additionally, to obtain a normative structural connectome, we used diffusion-weighted MRI acquisitions from 60 healthy volunteers (data set #2) from the human connectome project (HCP) database (db.humanconnectome.org; see Supplementary Table 2 for demographic details of data set #2). The healthy volunteers were not age-matched with the stroke cohort (mean age was 32, range 26–34; see Supplementary Table 2 for additional details).

#### Data set #1

Thirty-seven patients were recruited from the inpatient rehabilitation unit of the University Hospital of Geneva. Part of these data were published elsewhere.^20^ Inclusion criteria were (i) clinical diagnosis of stroke involving the territory of the middle cerebral artery as demonstrated by structural MRI and (ii) at least mild motor impairment (upper FMA of at most 55 points) at the beginning of rehabilitation. Sixty-three patients were included in the initial study. The selection of 37 patients (mean age was 65.6, range 28–85; see Supplementary Table 1 for additional details) for the present study has then been made on the basis of data completeness, which consists of the presence for each patient of (i) a diffusion-weighted and *T*_2_-weighted imaging obtained 2–4 weeks after the stroke for lesion segmentation and (ii) upper FMA at 2 weeks and 3 months after the stroke. Patients for which one of these elements was lacking have been excluded from the present group. All patients received standard therapy at the stroke unit during the acute phase and an individually tailored multidisciplinary rehabilitation programme in the subacute and chronic phases. All patients received two times 30 min of physical therapy per day on 5 days per week and five times 30 min of occupational therapy per week on an inpatient basis for 8–16 weeks, followed by outpatient treatment of 1–4 h of physical and occupational therapy per week.

All experiments were reviewed and approved by the Commission Cantonale d’Ethique de la Recherche de Geneve, Switzerland. Informed consent forms, including consent to share de-identified data, were collected for all subjects, and all methods were carried out in accordance to the Declaration of Helsinki.

### Clinical assessments

Trained physical or occupational therapists performed standardized clinical assessments of upper extremity motor function at 2–4 weeks and 3 months after stroke using the upper extremity items of the FMA^21,22^ with a maximal score of 66 points. We computed the FMA recovery score as $100\cdot \frac{\mathrm{FM}A_{3\mathrm{months}}-\mathrm{FM}A_{2\mathrm{weeks}}}{66-\mathrm{FM}A_{2\mathrm{weeks}}}$, where $\mathrm{FM}A_{3\mathrm{months}}$ and $\mathrm{FM}A_{2\mathrm{weeks}}\;$ are the FMA score at 3 months and 2 weeks post-lesion, respectively.

### MRI acquisition

For both data sets, standard anatomical images and diffusion-weighted images were acquired.

#### Data set #1

High-resolution structural T_2_-weighted (echo time/repetition time = 376 ms/5.0 s; voxel size = 0.45 × 0.45 × 0.90 mm^3^) volumes were obtained on a 3.0-T Siemens Trio TIM 3.0-T scanner using a 64-channel coil. Additionally, whole brain, single-shot echo-planar (EPI) diffusion-weighted volumes (30 non-collinear directions; *b* = 1000s/mm^2^; 64 slices; voxel size 1.8 × 1.8 × 2.0 mm^3^; echo time/repetition time = 82 ms/8.2 s; acquisition time = 4 min 40 s) plus one volume without diffusion weighting (*b* = 0s/mm^2^) were acquired parallel to a line intersecting the anterior and posterior commissure.

#### Data set #2

High-resolution structural MRI was acquired using a 3D MPRAGE *T*_1_-weighted (TR = 2400 ms, TE = 2.14 ms, TI = 1000 ms, flip angle = 8°, FOV = 224 × 224, voxel size = 0.7 mm isotropic). The following sequence was used for diffusion-weighted MRI: spin-echo EPI, TR = 5520 ms, TE = 89.5 ms, flip angle = 78°, FOV = 208 × 180, 3 shells of *b* = 1000, 2000 and 3000 s/mm^2^ with 90 directions plus 6 *b* = 0 acquisitions; total acquisition time was 9 min 50 s. HCP-minimally preprocessed images were used for all acquisitions.

### Lesion masking

*T*_2_-weighted and DWI sequences were used to delineate ischaemic lesions using the software MRIcro (http://www.cabiatl.com/mricro/). Afterwards, images and lesion masks of individual subjects were normalized to canonical Montreal Neurological Institute (MNI) space (2 × 2 × 2 mm resolution) using SPM8 software. Specifically, we used the normalize function in SPM8 with regularization type MNI, a non-linear frequency cut-off of 25 and 16 iterations for the non-linear warping. Lesions were masked during normalization to avoid distortions.^23^

### Streamline tractography connectomes

Part of the structural connectome used in this paper was already deployed in a previous publication.^24^ Diffusion-weighted scans of the healthy subjects (data set #2) were analysed using MRtrix3^25^ (http://www.mrtrix.org/) with the following operations: multi-shell multi-tissue response function estimation, constrained spherical deconvolution, tractogram generation with 10^7^ output streamlines.

### Brain connectivity measures

**Figure 1:**
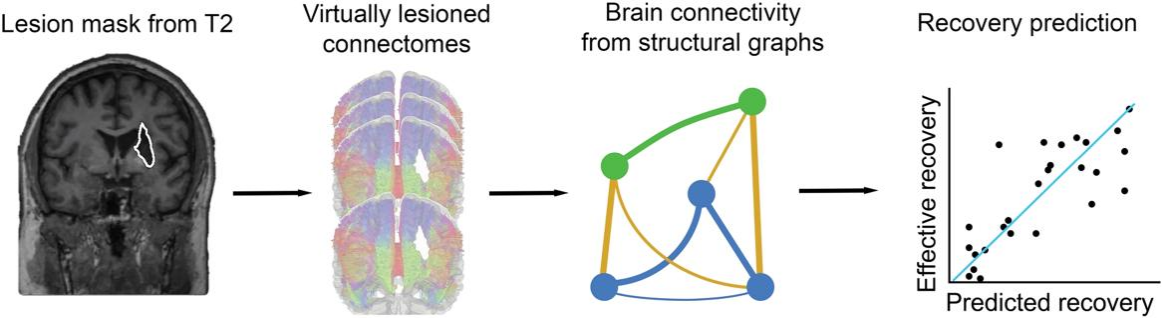
**Prediction model based on brain connectivity measures.** *First step:* a lesion mask is drawn for each patient from the structural MRI. *Second step:* for each patient, the lesion mask is intersected with each of the 60 healthy streamline tractography connectomes deleting all white matter tracts passing through the lesioned area leading to 60 virtually lesioned connectomes. *Third step:* from each of these virtually lesioned connectomes, we estimate brain connectivity measures and the latter are averaged over connectomes. *Fourth step:* these measures are used as input for a ridge regression model to predict motor improvement.

In order to compute a brain connectivity matrix per patient, we first virtually lesion each of the 60 healthy connectomes by intersecting them with each patient’s lesion mask using the tckedit function of MRtrix 3.0 software and deleting all white matter tracts passing through the lesioned area (Fig. 1). This indirect estimation of the stroke impact on the whole brain connectome is more readily available than direct measures of functional and structural connectivity obtained with MRI. For each patient, we thus obtained 60 virtually lesioned connectomes. Glasser’s multimodal cortical atlas^26^ converted to volume was split into the two hemispheres (first 180 areas on the left and last 180 on the right) and used to parcellate the cortex into *N* = 360 regions of interest and generate the *N × N* structural connectome using the *tck2connectome* function of MRtrix 3.0 software for each patient and each damaged connectome. For each impacted connectome, we then constructed a graph considering each brain area as a vertex and the number of fibres connecting two regions divided by the region volumes (sum of connected regions) as edges connecting two vertices. From these graphs, we computed seven brain connectivity measures considering the weights of the edges (i.e. weighted graph) and 10 measures on binary graphs (i.e. graphs with edges either 0 or 1; see below for details) using the Brain Connectivity Toolbox^27^ (see Supplementary Table 3 for a complete list of the measures considered and for their mathematical definitions and interpretations) (Fig. 1). Some measures are not represented by a single value for the whole network, but by one value for each of the 360 nodes. When this was the case, we considered both the mean and the median to obtain one global value. For each patient and each connectivity measure, we then averaged the 60 values obtained from the 60 virtually lesioned connectomes. To compute binary measures, the weighted adjacency matrices were first binarized through a thresholding process. As the threshold is inversely proportional to the density of the binarized graph, this must be chosen with care. If the density is too low, the graph is too sparse, and some points will be disconnected from the rest of the network. On the other hand, a too high density will lead to too many edges being present, which makes the interpretation of the graph patterns irrelevant. A threshold was chosen to obtain a density equal to the weighted density of the weighted matrices.

### Cortico-spinal tract asymmetry

Data set #1 diffusion-weighted MRI data were preprocessed using the DIFF_PREP and DIFF_CALC methods of Tortoise software.^28,29^ The tensors were then spatially normalized using the Diffusion Tensor Imaging ToolKit (DTI-TK).^30–32^ Mean fractional anisotropy (FA) values for left and right CST were obtained with FMRIB Software Library (FSL).^33–35^ The percentage of asymmetry was then computed using the Stinear’s formula:^36^


$$\mathrm{Asymmetry}=\frac{FA_{H}-FA_{L}}{FA_{H}+FA_{L}}$$

where FA_L_ is the mean FA value of the left (right) CST and FA_H_ is the mean FA value of the right (left) CST for a lesion in the left (right) hemisphere.

### Prediction models

In order to test whether brain connectivity measures will improve the prediction of recovery as compared to clinical state-of-the-art measures, we compared the predictive power of five different sets of features (Table 1). The first set of features was composed of immediately available information in the clinical setting: patient’s age and initial FMA impairment. In the second set of features, we added the lesion volume. The CST asymmetry value was then added to obtain the third set of features. This set of features is the one that so far in literature demonstrated the highest accuracy in predicting motor recovery.^10,11,37^ In the fourth data set, we added the brain connectivity measures to the other features. Due to the number of connectivity features included and to avoid collinearity issues in the training part of the prediction model, a feature selection step using variance inflating factors (VIF) was carried out to select the most important features. Finally, in the fifth set of features we excluded CST asymmetry (i.e. we considered only patient’s age, initial FMA impairment, lesion volume and brain connectivity measures). Indeed, CST asymmetry requires advanced imaging acquisitions (as diffusion-weighted MRI) that are not always available in clinical setting. Also, for the fifth set of features, the most predictive brain connectivity measures were selected using VIF.

**Table 1:** List of sets of features tested

Models/Features	Age + Initial FMA	Volume	CST asymmetry	Brain connectivity
Set 1	x			
Set 2	x	x		
Set 3 (Benchmark)	x	x	x	
Set 4	x	x	x	x
Set 5	x	x		x

For each set, features were standardized to obtain a mean of 0 and a standard deviation of 1 and then used as input for a ridge regression model to predict FMA recovery score (Fig. 1).


$$\mathrm{Ridge}\;\mathrm{regression}:\;\mathrm{mi}n_{\beta }\left\{\frac{1}{N}y-X\beta_{2}^{2}+\lambda_{2}\beta_{2}^{2}\right\}$$

The standardization, feature selection and prediction steps were included in a leave-one-subject-out (LOO) cross-validation approach. For the fourth and fifth sets of features, VIFs were used in each LOO fold to iteratively exclude the most collinear feature until a certain VIF threshold was reached. A range of VIF thresholds were tested (every unit from 5 to 200). In each fold, the regularization *λ*_2_ coefficient of the ridge regression was optimized by identifying a value that minimized LOO prediction error over the training set. Optimal weights were solved across the entire training set using a grid search to minimize error for the ridge regression equation by varying lambda. These model weights were then applied to the left-out fold (i.e. subject) to predict the behavioural score. Model accuracy was assessed using the coefficient of determination: $R^{2}=1-\frac{\sum \;{\left(Y-Y^{\prime }\right)}^{2}}{\sum \;{\left(Y-\bar{Y^{\prime }}\right)}^{2}}$, where *Y* are the measured FMA recovery score, $Y^{\prime }$ are the predicted FMA recovery score and $\bar{Y^{\prime }}$ is the mean of predicted FMA recovery scores. We then compare each set of features based on their accuracy. As stated in the Introduction, predicting the FMA recovery and using the initial FMA score as part of the independent variables come with some limitations in the form of ceiling effects and mathematical coupling. These limitations may lead to the observation of a spurious correlation between the initial impairment and the recovery even if the correlation between the initial impairment and outcome is null. The presence of this issue in our analysis would lead to an overestimation of the goodness-of-fit of our model. Fortunately, this issue can be ruled out by observing a similar goodness-of-fit using the same model to predict stroke outcome (i.e. FMA value at 3 months post-stroke) instead of recovery.^5^ For this reason, we tested the ability of our best model to predict outcome using the same model reported above (i.e. ridge regression model with LOO approach).

### Classification task

In order to demonstrate the classification ability, i.e. ability to obtain a categorial classification of the patients (0–1) of the five different sets of features, we divided the patients in fitters and non-fitters following the proportional recovery rule as in Koch *et al.*^19^ Specifically, fitters/non-fitters are patients that follow/not the proportional recovery rule. It has been demonstrated that this separation into two classes is robust and not an artefact of mathematical coupling, as it was present also when using Bayesian modelling.^8^ In order to determine these two groups of patients, we performed a hierarchical clustering using Spearman correlation distance on the values of FMA points that should be recovered following the 70% proportional recovery model versus the actual number of FMA points effectively recovered by each patient. We additionally used a simple threshold of 30% FMA recovery score to separate fitter from non-fitters, based on literature,^20^ and we confirmed that this led to precisely the same labelling as obtained from the hierarchical clustering method. Once we obtained this classification of the patients, we compared each set of features based on their ability to distinguish between fitter and non-fitter using specificity, sensitivity, positive predictive value (PPV) and negative predictive value (NPV).

### Statistical analysis

To estimate the correlation of our measures with FMA recovery score, we computed Spearman correlation values between FMA recovery score and each benchmark feature (initial impairment, lesion volume, age and CST asymmetry) and each brain connectivity feature. The associated *P*-values were Bonferroni corrected for the number of features tested. To assess the amount of shared information between features, we computed Spearman correlation values for each pair of variables. Furthermore, we performed hierarchical clustering based on the Spearman correlation distance to identify clusters of variables sharing similar information. We have also assessed the ability of the features to individually distinguish between fitter and non-fitters by performing Wilcoxon rank–sum tests.

Additionally, we have bootstrapped 95% confidence intervals for each prediction model to assess the statistical difference between Set 4 and the other feature sets. Specifically, we have randomly selected (*N* = 10 000) 32 points predicted by the model and computed their *R*^2^. Then, we derived 95% confidence intervals by computing the 2.5 and 97.5 percentiles of the obtained distribution.

## Results

### Brain connectivity measures correlated with motor impairments

We first assessed which features were most predictive of recovery by computing Spearman correlation with the FMA recovery score, both for benchmark features and brain connectivity measures. As expected, when considering the features already known to be predictive of recovery, we found that initial impairment (ρ = 0.68, *P*-value < 0.00001) and CST asymmetry (ρ = −0.71, *P*-value < 0.01) correlated the most with the recovery. Lesion volume (ρ = −0.49, *P*-value = 0.03) had a strong correlation too, but age (ρ = 0.03, *P*-value = 0.88) was a poor predictor in our data set. Importantly also both binary and weighted brain connectivity measures showed a strong correlation with motor improvement. Specifically, we found that density (ρ = 0.53, *P*-value < 0.01 Bonferroni corrected), global efficiency (ρ = 0.55, *P*-value < 0.01), median degree (ρ = 0.48, *P*-value < 0.05), algebraic connectivity (ρ = 0.49, *P*-value <0.05), mean eigenvector centrality (ρ = 0.52, *P*-value < 0.05) and participation coefficient (ρ = 0.51, *P*-value ≤ 0.05) measures correlated with FMA recovery score (see Table 2). Additionally, these measures significantly distinguished fitters and non-fitters, as shown by their Wilcoxon rank–sum test *P*-values. Overall, poor recovery was thus observed when strokes hit highly connected brain areas (hubs) leading to a reduced global efficiency of information exchange on the whole network and between hubs.

**Table 2:** First column: correlation between all the features used (benchmark and brain connectivity measures) and FMA recovery score

	Property	Correlation value	Wilcoxon rank–sum test	
			U statistic	P-value
Benchmark features	Initial impairment	0.68**	5.50	<10^−5^
	Age	0.03	0.46	0.65
	Volume	−0.49**	−2.19	0.03
	CST asymmetry	−0.71**	−2.86	<10^−2^
Binary graph measures	Density	0.53**	2.29	0.02
	Median degree	0.48*	2.07	0.04
	Mean clustering coefficient	0.47	2.32	0.02
	Median clustering coefficient	−0.26	−0.96	0.33
	Mean flow coefficient	0.36	1.52	0.13
	Transitivity	−0.26	−0.91	0.36
	Modularity	−0.02	0.18	0.85
	Mean eigenvector centrality	0.52*	2.13	0.03
	Median eigenvector centrality	0.33	1.49	0.14
	Mean betweenness centrality	−0.20	−0.61	0.54
	Median betweenness centrality	0.11	0.55	0.58
Weighted graph measures	Algebraic connectivity	0.49*	2.19	0.03
	Mean eigenvector centrality	0.47	2.10	0.04
	Median eigenvector centrality	0.41	1.80	0.07
	Modularity	0.14	0.76	0.45
	Global efficiency	0.55 **	2.59	0.01
	Participation coefficient	0.51 *	2.26	0.02
	Quasi-idempotence	0.15	−0.09	0.93

For the reported features, (*) and (**) indicate correlation *P*-value Bonferroni corrected <0.05 and <0.01, respectively. Second column: *U*-statistic for the Wilcoxon rank–sum test between fitters and non-fitters. Third column: *P*-value for the Wilcoxon rank–sum test between fitters and non-fitters.

**Figure 2:**
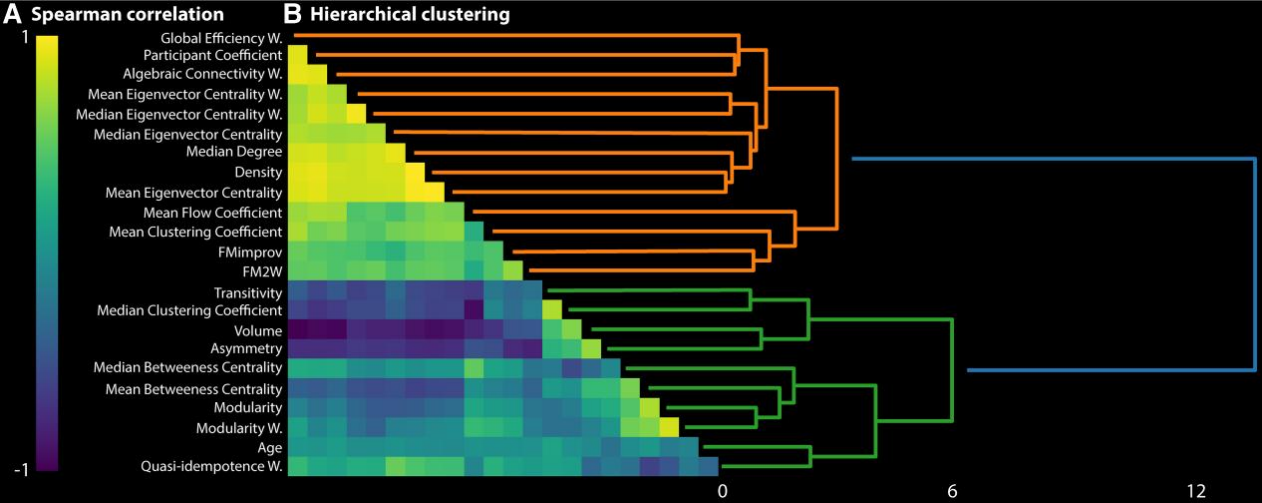
**Collinearity between predictive measures.** (**A)** Spearman correlation matrix of all included variables. Yellow indicates a positive and blue a negative correlation. (**B)** Hierarchical clustering based on the Spearman correlation matrix.

We then performed hierarchical clustering based on Spearman correlation to determine which brain connectivity measures and benchmark features share a high proportion of information (Fig. 2). Interestingly, the first clustering split divided the variables into two groups, and for both groups we had both measures of segregation and measures of centrality highlighting the importance of these two groups of features. However, the first one, which contained the benchmark features except the initial impairment, included global (i.e. over the entire network) brain connectivity measures such as modularity, transitivity and mean and median betweenness centrality. The second group, alternatively, contained measures of modularity and centrality on the level of individual nodes such as the participation coefficient and the mean and median of eigenvector centrality. These results might suggest that initial impairment correlate more with changes in local nodes, whereas motor improvement and CST asymmetry have a stronger influence on global changes. Finally, the strong correlation between brain connectivity measures further supports the need of a feature selection step before the prediction to reduce collinearity. This selection is based on the identification of VIF thresholds, and its results are described below.

### Only a limited number of patients fitted within the proportional recovery model

**Figure 3:**
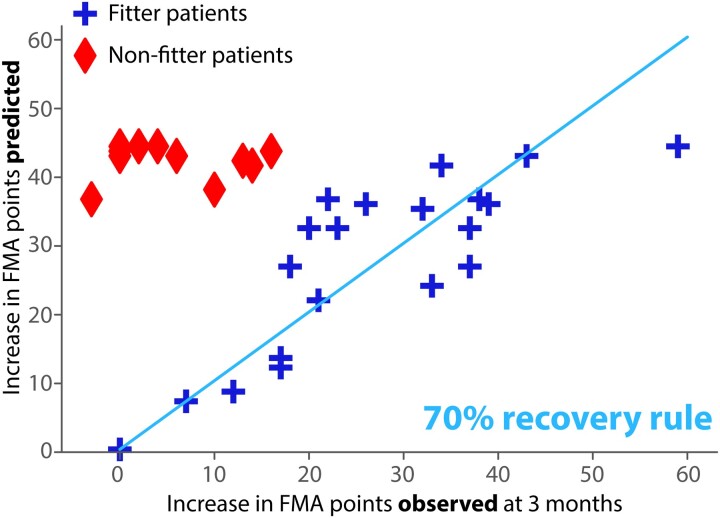
**Scatter plot showing fitter (cross symbols ) and non-fitter (rhomboid symbols) patients according to the proportional recovery model.** The separation into two recovery groups is visible. Patients that recovered more than 30% threshold of FMA recovery score coincided with the fitter patients, whereas the patients that recovered less than 30% threshold of FMA recovery score coincided with the non-fitter group of patients. The line represents a simple linear regression including the fitter patients.

Following the hierarchical clustering method, 45.9% of all patients (17/37) were determined to be fitters of the proportional recovery model, and 54.1% (20/37) were identified as non-fitters (Fig. 3). These results supported previous findings that only a limited portion of patients fit within the rule of the 70% of recovery (i.e. patients recover on average 70% of their initial impairments), demonstrating the urge of novel more precise predictive models. The proportion of non-fitters was important. This was largely due to the proportion of severe initial impairment (as defined by an FMA score at 2 weeks ≤20) in our set of patients. Indeed, 76% (28/37) (Supplementary Fig. 1) of all patients had a severe initial impairment, which was associated with greater odds of belonging to the non-fitters group.^19^ We also separated patients based on a 30% threshold of FMA recovery score, and we verified that the two resulting groups coincided perfectly with the fitter/non-fitter groups. Therefore, from now on, we will use this threshold to assess whether the prediction models correctly classify the patients.

### Brain connectivity measures significantly increased the accuracy of prediction

Correlation analyses as the ones reported above do not necessary imply that these features will be predictive of patients’ recovery. To test prediction and in particular to test whether brain connectivity measures would lead to a better prediction than benchmark features so far utilized (i.e. patient’s age, initial FMA impairment, lesion volume and CST asymmetry), we built five different linear regressor models using five different set of features (Table 1).

**Figure 4:**
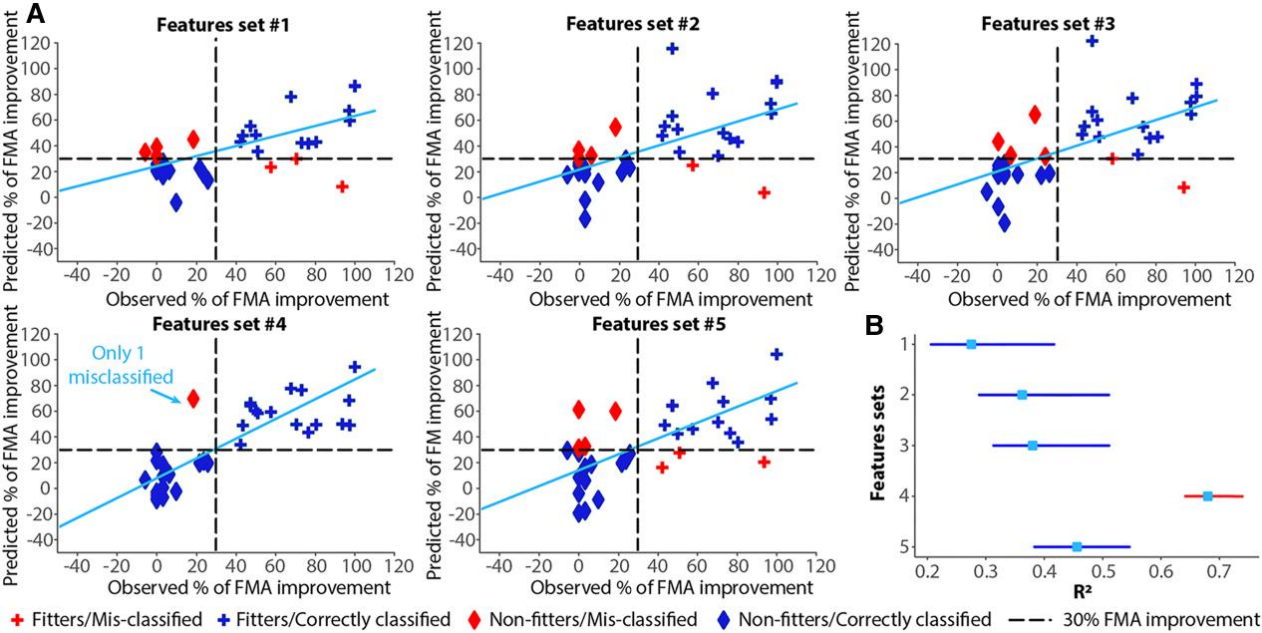
**Prediction accuracy.** (**A**) scatter plot of observed percentage of FMA recovery score versus predicted percentage of FMA recovery score for the five features set tested. In red the misclassified patients and in dark blue the correctly classified patients. The lines represent simple linear regressions including all participants. (**B**) Lines represent 95% confidence intervals for the proportional recovery prediction models *R*^2^ using bootstrapping for the five feature sets. Square indicates *R*^2^. Feature sets are significantly different if 95% confidence intervals do not overlap.

As expected from previous work, clinical and demographic features (i.e. patient’s age and initial FMA impairment) led to good prediction (*R*^2^: 0.27, Table 3 and Fig. 4A). The prediction could be further improved when lesion size (*R*^2^: 0.36) and the degree of CST damaged (*R*^2^: 0.38) were added. Importantly, when using the set of features that included on top of the benchmark features the brain connectivity measures, the predictive model significantly outperformed all the others in terms of accuracy (*R*^2^: 0.68, Fig. 4B and Supplementary Table 5). Finally, when removing CST asymmetry, which is rarely available, the prediction was still higher than benchmark features alone (*R*^2^: 0.46), though this difference was not statistically significant. The similarity in *R*^2^ between Set 3 and Set 5 demonstrates that CST asymmetry, which needs long and tedious DTI analyses, is equivalent to a full-brain analysis of the impact of the lesion on the network, which merely needs a binary image of the stroke lesion. Increased accuracy of prediction could instead be achieved only combining CST asymmetry and whole-brain network changes. Importantly, we obtained a similar accuracy (*R*^2^: 0.69) when using the brain connectivity measures on top of the benchmark features to predict outcome instead of recovery, thus ruling out the risk that our performance was spuriously inflated by mathematical coupling between the FMA score at baseline and the FMA recovery score.

**Table 3:** *R* ^2^, specificity, sensitivity and positive and negative predictive value for proportional recovery from the five sets of features

Features	R^2^	Specificity	Sensitivity	Positive predictive value	Negative predictive value
Set 1	0.27	0.82	0.76	0.81	0.78
Set 2	0.36	0.88	0.76	0.87	0.79
Set 3 (benchmark)	0.38	0.88	0.76	0.87	0.79
**Set 4**	**0**.**68**	**1**.**00**	**0**.**94**	**1**.**00**	**0**.**94**
Set 5	0.46	0.82	0.76	0.81	0.78

Highlighted in bold the set of features with the best prediction.

Importantly, when predicting whether a patient will recover according to the ‘proportional rule’ (i.e. more than 30% of the initial FMA value, so-called fitters), we observed a very high accuracy (specificity = 1.00, sensitivity = 0.94) with Set 4, which includes brain connectivity measures in addition to the benchmark features (Table 3 and Fig. 4). Overall, these results demonstrate that information about widespread network dysfunctions are pivotal for an accurate prediction of the patients’ recovery.

### Features selected and weights of the prediction

**Figure 5:**
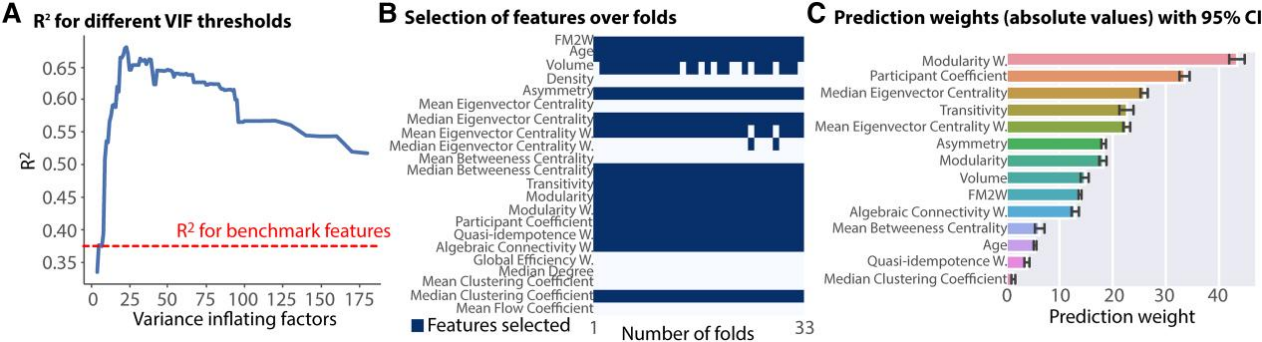
**Stability of global measures.** (**A)***R*^2^ versus VIF for Set 4. The dash line indicates the *R*^2^ for the benchmark features (Set 3). (**B)** Number of times a feature is selected per fold. (**C**) Weights fitted for each feature (average and standard deviation over folds).

Because of the high collinearity between connectivity features, the number of features included in the prediction model was reduced using VIF in each of the leave-one-out folds both for Sets 4 and 5. In both cases, the predicting power increased with the reduction of the number of features up to a maximum (VIF = 23, number of brain connectivity features = 10). Yet, the predicting power for Set 4 was higher than the benchmark features for all considered VIF thresholds (where the maximum VIF threshold corresponds to a number of 14 brain connectivity features) highlighting the consistency over features on increasing the prediction (Fig. 5A). Additionally, the selected brain connectivity features were similar between Sets 4 and 5 highlighting the stability of the predictive network’s characteristics.

In order to further assess the stability of the predictive connectivity features, we counted how many times a feature was selected over folds. Importantly, the selection was highly consistent over subjects highlighting the generalizability of our approach to unseen patients and novel data sets (Fig. 5B). Indeed, only the mean eigenvector centrality and the lesion volume were not selected at every fold (i.e. 2- and 8-folds for mean eigenvector centrality and lesion volume, respectively). This shows that some brain connectivity features are very collinear with the lesion volume and carry the same information. Interestingly, some of the features that showed a strong correlation with FMA recovery score such as density, global efficiency and median degree were not selected in the prediction model, further highlighting that correlation does not necessarily imply features to be predictive of patients’ recovery. Vice versa, some of the features with low correlation, such as modularity, had a strong predictive information. Indeed, we extracted the weights fitted for each feature and averaged them over the folds (Fig. 5C). Interestingly, the features with highest weights (i.e. higher than benchmark features) belonged to both clusters of the first split of the hierarchical clustering and were measures related to both segregation (modularity weighted, participant coefficient, transitivity) and centrality (median eigenvector centrality and mean eigenvector centrality weighted). These two groups of measures describe how well a network can be subdivided into subnetworks (modules) and how each node influences the network, respectively. Importantly, whereas modularity and transitivity are global measures, participant coefficient and eigenvector centrality are calculated on the level of individual nodes. This suggests that both local and widespread changes within the network consequently to a stroke are important characteristics to consider for an accurate prediction of recovery.

## Discussion

Personalized outcome and recovery prediction are one of the main foci of stroke research. Indeed, in recent years, novel evolving therapeutic options have revolutionized post-stroke treatment. However, even experienced clinicians find it difficult to accurately predict patients’ level of recovery^2^ and, consequently, to assign them to the appropriate treatment. For this, several prediction models have been proposed in the last decades. The majority of them utilized clinical and demographic information to predict motor recovery at 3 months post-lesion, i.e. the moment at which the majority of the recovery has occurred.^38^ More recent studies included measurements obtained by neuroimaging or neurophysiological recordings that better capture interindividual lesion-induced neural abnormalities, leading to improved prediction accuracy when combined with clinical and demographic information.^10,16–19,39–44^ However, these neuro-biomarkers are often obtained by special imaging acquisitions (e.g. functional MRI or diffusion-weighted imaging) that require computationally expensive algorithms of analysis and prediction, thus posing several challenges for practical application in clinical routine. Here we proposed a novel computationally efficient (i.e. 10 min on a recent desktop computer—12 cores AMD Ryzen 5900×—for the predictions starting from the lesion mask) prediction model that leverages high-quality tractography data from a public data set in healthy individuals and combines them with the patient’s lesion mask without the need for diffusion-weighted MRI acquisitions. Our model outperformed state-of-the-art methods both in term of specificity and sensibility. Here we discuss these findings with an emphasis on the additional knowledge about mechanisms driving functional recovery garnered with our prediction model and the clinical advantages of our approach.

### Whole brain connectivity measures predict motor recovery better than cortico-spinal tract integrity on its own

We considered five different models with different sets of features (**Table 1**), and we compared both actual prediction in terms of *R*^2^ and binary, categorial (0–1) follow-up outcomes (i.e. in this case 0 represents a poor recovery—less than 30% of the initial impairment). Indeed, while the coefficient of determination gives a better estimation of the predictive power of a model, binary outcomes such as favourable versus unfavourable motor recovery are often more informative in clinical practice.^39^ We first considered only age and initial motor impairment that have been extensively studied, e.g. in the Acute Stroke Registry and Analysis of Lausanne (ASTRAL), which is so far the model tested in the largest data sets (i.e. more than 10 000 patients from different countries and continents).^45^ When considering these features, we obtained low predictive power similar to previous studies (*R*^2^ = 0.27), which highlights that information about initial deficits is not enough to precisely predict changes over time. Prediction was improved when considering lesion volume and CST asymmetry as also previously shown. For instance, Byblow *et al.*^41^ showed that the change in FMA score was predicted by initial FMA score, presence of motor-evoked potentials and FA asymmetry of the CST. These results were further validated in a recent study from Lin *et al.*,^10^ which utilizes a template built from MRI acquisitions from a group of healthy subjects to estimate CST asymmetry. Importantly, our model using benchmark features (i.e. patient’s age, initial FMA impairment, lesion volume and CST asymmetry) explained a comparable proportion of variance to the model showed by Lin *et al*. However, the anatomy of the damage might be different in each patient, and so even if two subjects match for initial severity, they might present different recovery trajectories. While the analysis of fibre tracts integrity can help elucidating these differences, the choice of the CST entails two important drawbacks. It first limits the analysis to only the main pathway involved in motor control neglecting the influence from secondary pathways that are also important for recovery;^46–48^ and, more importantly, it requires the analysis of individual diffusion-weighted images. Here we overcome these limitations by intersecting the patient’s lesion mask with 60 healthy high-resolution streamline tractography connectomes and computing whole brain connectivity measures. When brain connectivity measures were considered with age, initial impairments and lesion volume, they obtained performance similar to the benchmark features, demonstrating that whole brain measures are equivalent to characteristics related to the integrity of the CST. Importantly, the brain connectivity measures, combined with CST integrity measures, outperformed all other models.

The use of embedding patients’ lesions into a tractography atlas computed from a large population of healthy subjects^49^ has already been recognized for its unique potential (e.g. shortlisted for the 2019 Nature Research Award for Driving Global Impact^18,50,51^). Indeed, the streamline tractography connectome from a large population of healthy subjects^49^ or of the 60 healthy subjects used in our study has very high spatial resolution (i.e. 1 mm isotropic), which allows to better capture interindividual variability. Similar spatial resolution in single patients would require very long and clinically impracticable diffusion-weighted MRI acquisitions. The virtual lesion overcomes this problem at the computational cost for the clinician to manually draw the lesion mask, which can be considerably time-consuming and requires some expertise. Yet novel deep neural networks are continuously being proposed to automatize or semi-automatize this process and consequently significantly reduces the timing and the expertise needed to obtain the lesion masking. Importantly, the tractography atlases computed from a large population of healthy subjects require averaging over subjects of brain connectomes. In our case, instead, the averaging step is performed at the level of the brain connectivity measures. It is interesting to notice that both approaches lead to accurate estimation of brain changes after lesion and consequently to accurate prediction of recovery.

### Post-stroke motor symptoms are driven by local and global alteration

Importantly, for all our models, the pipeline comprised a ridge regression model and leave-one-patient-out cross-validation. The latter proves generalization of our models to unseen patients and hint to the usability of these models for personalized outcome prediction, which is in line with the objective of precision medicine.^39^ The ridge regression model was motivated by our interest in identifying the discriminative power of each feature to infer possible mechanisms of neural recovery. Indeed, while more advanced machine learning models, such as (deep) neural networks, nearest neighbour algorithms, random forests or kernel support vector machines often yield higher predictive power, they are denoted as ‘black-box’ models because they do not allow identifying the weight of individual features. Importantly, we demonstrated that more advance models, such as elastic net, Bayesian Ridge, etc., still yielded predictive power similar to ridge regression model (Supplementary Table 4).

The most discriminative brain connectivity features were modularity weighted, participant coefficient, transitivity, median eigenvector centrality and mean eigenvector centrality weighted. These are measures of segregation (i.e. measures of how a network can be split into more efficient local networks) and of centrality (i.e. measures of the influence of a node in a network). This suggests that a preserved ability of the network to integrate and segregate in subnetworks is pivotal for the recovery. Previous results already hinted to a possible reduction in modularity and efficiency of information transfer, which could manifest in the form of aberrant durations of functional networks^52–55^ or in decreased static functional connectivity.^56^ Yet the indirect quantification of this efficiency alteration from simple lesion maps is easier and faster to implement in clinical practice than measures extracted from functional resting-state or diffusion-weighted MRI acquisitions. Additionally, a single node in the network can strongly influence neural plasticity and clinical recovery. Indeed, as also demonstrated by the high correlation between density and motor improvement, a small stroke that hits well-connected hubs will lead to a poorer recovery than a stroke that hits unconnected hubs. Importantly, these differences are not captured by benchmark features. Indeed, lesion volume and CST asymmetry will not be able to distinguish between a small stroke that has or has not hit well-connected hubs. Additionally, whereas modularity and transitivity are calculated over the entire network; participant coefficient and eigenvector centrality are calculated on the level of individual nodes. This suggests that, as hypothesized, changes consequently to a stroke occurred both locally tied to the lesion and in widespread highly connected brain areas, and both these changes need to be considered for an accurate prediction of recovery. Therefore, our results support recent works that went beyond focal lesions and incorporated measures of network changes in the brain, based on the realization that stroke impacts the entire network.^14,40,42^ In the same direction, recent investigations showed better discrimination of patients when using full electroencephalography (EEG) topography of responses as compared to single EEG electrodes located in proximity of the lesion, because the former captures changes over several cortical regions, extending beyond the location of the damage.^57–59^ Yet similar to functional MRI, EEG recordings require long and tedious acquisitions and computationally costly algorithms for the analysis. Finally, importantly, the same brain connectivity measures allowed an accurate recovery prediction also when holding out the CST asymmetry, further demonstrating that local changes due to the lesion can be accounted for using characteristics of CST integrity or brain connectivity measures. However, because of the highest simplicity of the brain connectivity measure, the latter are of highest clinical value.

### Towards a clinically effective model

We showed that brain connectivity features allow remarkable prediction accuracy with a relatively low computational effort, which is a sine qua non characteristic for clinical usability and statistically significant higher accuracy when combined with pre-existing measures of CST integrity. In particular, our best model (benchmark features combined with brain connectivity measures) significantly improves predictions of non-fitters, i.e. patients that do not experience proportional recovery. Lack of fitting to the 70% rule seems to be particularly problematic for patients with severe baseline motor deficits (i.e. FMA score at 2 weeks ≤ 20).^19^ Importantly, these patients are those that do not show satisfactory improvements, thanks to current rehabilitative intervention, and experience chronic clinical deficits.^37^ Therefore, our brain connectivity method would be particularly beneficial to predict recovery of moderate to severe patients to further improve therapeutic choice and hopefully motor outcome. However, for this, in future, our model will need to be extended to predict motor recovery in relation to the assigned therapeutic protocol. This will be pivotal to correctly stratify patients within treatment options.

It is important to highlight that at the moment, the main computational bottleneck in our approach is the drawing of the lesion mask. However, many groups are developing novel deep neural networks to automatize or semi-automatize this process and consequently significantly reduce the timing to obtain the lesion masking. Importantly, while in our model the lesions were drawn from structural MRI acquired around 2 weeks post-stroke, computational tomography (CT) scans obtained at hospitalization could be used to identify the region affected by the stroke. This will further improve the clinical usability of our method because CT scans, as compared to MRI, are routinely obtained for stroke patients in many clinical settings. Yet further studies are necessary to demonstrate that lesion masks from CT scans can be used in our method. Additionally, in future works, our results would need to be replicated in a larger cohort and, ideally, in two different cohorts for the training and testing steps. Indeed, the current main limitation of our study is the low number of participants. For this, data acquired in clinical settings without specific scientific purposes may enable a better representation of the full spectrum of stroke patients and thus allow for a better validation.^39^ Importantly, because our model only utilizes lesion masks obtained from MRI, our method would be easily extendable to such data sets and so easily translatable to clinical practice.

Finally, importantly, our model will have to be extended to other post-stroke symptoms. Indeed, although motor impairment represents one of the main post-stroke symptoms (frequencies as high as 50% and 80%^1^), deficits also concern other behavioural domains such as language, attention and working memory.^60^ Moreover, a large fraction of strokes affects subcortical structures, especially white matter tracts, either exclusively or in addition to cortical lesions leading to the simultaneous presence of deficits in more than one behavioural domain.^60^ We expect that our brain connectivity measures will generalize to other symptoms. Indeed, a few recent studies demonstrated that EEG graph measures and particularly small-worldedness characteristics (a measure of the balance between local connectedness and global integration of a network, representing the brain network organization) correlated with functional recovery measured in activities of daily living.^61–63^

## Supplementary Material

fcad055_Supplementary_Data

## Data Availability

Further information and requests for resources should be directed to and will be fulfilled by the Lead Contact, Dr. Elvira Pirondini. Codes and processed data generated in this study will be available on GitHub (released upon article publication).

## Abbreviations

ASTRAL =: Acute Stroke Registry and Analysis of Lausanne
CST =: cortico-spinal tract
DTI-TK =: Diffusion Tensor Imaging ToolKit
EPI =: echo-planar
FA =: fractional anisotropy
FMA =: Fugl–Meyer Assessment
FSL =: FMRIB Software Library
HCP =: human connectome project
LOO =: leave-one-subject-out
MNI =: Montreal Neurological Institute
NPV =: negative predictive value
PPV =: positive predictive value
VIF =: variance inflating factors
