# Supplementary material for: Prediction of post-stroke motor recovery benefits from measures of sub-acute widespread network damages
Source: Brain Commun. 2023 Mar 1;5(2):fcad055. doi: 10.1093/braincomms/fcad055 (PMC10016810; doi:10.1093/braincomms/fcad055)

**SUPPLEMENTARY MATERIAL**

**Supplementary table 1:** Demographic information for dataset #1.

| Age (Mean±SD) | Sex | Hand | Lesion Side | FMA at 2 weeks (Mean±SD) | FMA at 3 months (Mean±SD) |
| --- | --- | --- | --- | --- | --- |
| 66±12 years | 17 women | 34 right-handed | 19 left, 18 right | 17±18 | 33±24 |

**Supplementary table 2:** Demographic information for dataset #2.

| Age by bracket | Sex | Hand |
| --- | --- | --- |
| [31-34] 54  [26-30] 6 | 36  women | 34 right-handed |

**Supplementary table 3:** List of brain connectivity measures and their meaning.

| **Category** | **Measure name** | **Meaning** |
| --- | --- | --- |
| Basic Measures | **Degree** | The total number of edges connected to a particular vertex. A node with a high degree is called a *hub*. |
|  | **Density** | The number of connections a network has, divided by the total possible connections a network could have. A high density would mean that a network is highly connected. |
| Global Measures | **Global efficiency** | A high global efficiency is associated with an efficient information transfer in the network. |
|  | **Algebraic connectivity** | A high algebraic connectivity indicates a high difficulty to partition a graph x. |
| Measures of segregation | **Clustering coefficient** | Clustering coefficient is a property of individual nodes in a network. It tells how well connected the neighborhood of a node is. If the neighborhood is fully connected, the clustering coefficient is 1 and a value close to 0 means that there are hardly any connections in the neighborhood. |
|  | **Transitivity** | The overall probability for a network to have adjacent nodes interconnected, thus revealing the existence of tightly connected communities (or clusters, subgroups, cliques) |
|  | **Modularity** | A high modularity indicates the partition subdivides the network into clearly delineated modules. |
|  | **Participation coefficient** | It’s a measure of modularity on the level of individual nodes. Participation coefficients measure the distribution of a node's edges among the communities of a graph. If a node's edges are entirely restricted to its community, its participation coefficient is 0. |
| Measures of centrality | **Betweenness centrality** | For every pair of vertices in a connected graph, there exists at least one shortest path between the vertices such that either the number of edges that the path passes through (for unweighted graphs) or the sum of the weights of the edges (for weighted graphs) is minimized. |
|  | **Flow coefficient** | High flow coefficient indicates low resistance to the passage of flow. Similarly, low coefficient indicates higher resistance to the passage of flow and high pressure drop for a given flow. |
|  | **Eigenvector centrality** | A high eigenvector score means that a node is connected to many nodes who themselves have high scores. |

**Supplementary Table 4:** R^2^, specificity, sensitivity, and positive and negative predictive value for different prediction models on the set of features 4 with the best VIF threshold. PPV: positive predictive value; NNV: negative predictive value.

| **Value** | **Ridge Regression** | **Multilinear Regression** | **Lasso** | **Elastic Net** | **Bayesian Ridge** |
| --- | --- | --- | --- | --- | --- |
| **R^2^** | 0.68 | 0.68 | 0.67 | 0.66 | 0.51 |
| **Specificity** | 1.00 | 1.00 | 1.00 | 1.00 | 0.94 |
| **Sensitivity** | 0.94 | 0.94 | 0.94 | 0.94 | 0.82 |
| **PPV** | 1.00 | 1.00 | 1.00 | 1.00 | 0.93 |
| **NPV** | 0.94 | 0.94 | 0.94 | 0.94 | 0.84 |

**Supplementary Table 5:** 95% confidence intervals for the proportional recovery prediction models R^2^ using bootstrapping.

| **Feature set** | **Lower bound** | **Original r^2^** | **Upper bound** |
| --- | --- | --- | --- |
| **Set 1** | 0.206 | 0.275 | 0.417 |
| **Set 2** | 0.288 | 0.362 | 0.511 |
| **Set 3** | 0.313 | 0.380 | 0.511 |
| **Set 4** | 0.640 | 0.680 | 0.741 |
| **Set 5** | 0.383 | 0.456 | 0.546 |

**Supplementary Figure 1:** Histogram with 20 bins of the upper FMA score at 2 weeks in dataset #1 (37 patients total). In the y-axis the number of participants and the x-axis the FMA score at 2 weeks.


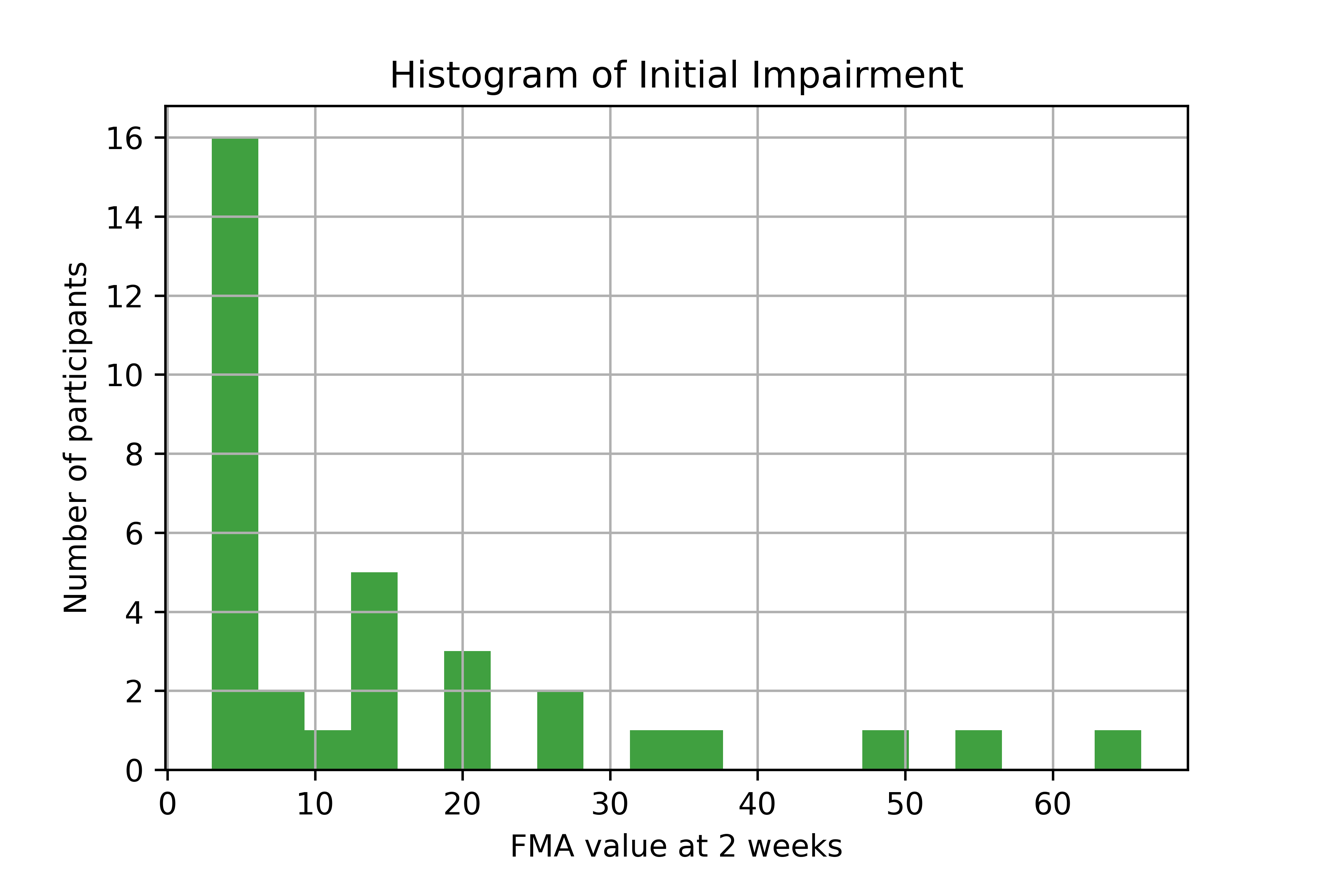

Supplement: fcad055_Supplementary_Data [file fcad055_supplementary_data.docx]
